# Assessment of Exenatide Extended‐Release for Maintenance of Diabetic Remission in Cats

**DOI:** 10.1111/jvim.70069

**Published:** 2025-03-19

**Authors:** Chen Gilor, Linda M. Fleeman, Sean E. Hulsebosch, Stijn J. M. Niessen, Charlotte R. Bjørnvad, Jully Pires, Katarina Hazuchova, Jocelyn Mott, Allison L. O'Kell, Ruth Gostelow, Adam J. Rudinsky, Audrey K. Cook

**Affiliations:** ^1^ Department of Small Animal Clinical Sciences College of Veterinary Medicine, The Ohio State University Columbus Ohio USA; ^2^ Department of Veterinary Medicine and Epidemiology University of California, Davis Davis California USA; ^3^ Department of Small Animal Clinical Sciences University of Florida, College of Veterinary Medicine Gainesville Florida USA; ^4^ Animal Diabetes Australia Collingwood Victoria Australia; ^5^ Royal Veterinary College Hatfield Hertfordshire UK; ^6^ Veterinary Specialist Consultations & VIN Europe Hilversum the Netherlands; ^7^ Department of Veterinary Clinical Sciences Faculty of Health and Medical Sciences, University of Copenhagen Frederiksberg Denmark; ^8^ Small Animal Clinic, Internal Medicine Justus‐Liebig‐University Giessen Giessen Germany; ^9^ Department of Small Animal Clinical Sciences College of Veterinary Medicine and Biomedical Sciences, Texas A&M University College Station Texas USA

**Keywords:** diabetes mellitus, feline, GLP‐1, glucagon, remission, weight loss

## Abstract

**Background:**

Insulin‐treated diabetic cats frequently achieve transient remission. The glucagon‐like peptide‐1 receptor agonist, exenatide extended‐release (exenatide‐ER), preserves β cell function in people with type 2 diabetes mellitus (DM).

**Objectives:**

Investigate the effect of exenatide‐ER on the duration of diabetic remission in cats.

**Animals:**

Twenty‐two client‐owned cats with recent diabetic remissions.

**Methods:**

Placebo‐controlled, single‐blinded study. Cats were assigned randomly to receive exenatide‐ER (0.13 mg/kg) or saline injection SC, once monthly for 2 years or until DM relapsed. Cats were fed low‐carbohydrate diets; weight control was actively supervised. Paired *t*‐tests and Mann–Whitney were used to compare pre‐ versus post‐study characteristics within groups and between group outcomes, respectively.

**Results:**

Treatment groups (placebo, *N* = 10; exenatide‐ER, *N* = 12) were similar in age, sex, and body weight upon inclusion. Thirteen cats completed the 2‐year study without diabetic relapse. Nine cats (placebo, *n* = 4; exenatide‐ER, *n* = 5) exited prematurely. Three of these exited because of DM relapse (placebo: *N* = 1, day 212; exenatide‐ER: *N* = 2, days 553 and 558). There was no difference in remission duration between treatments (placebo: 669 [121–721]; exenatide‐ER: 662 [28–735] days, *p* = 0.9). Median body weight decreased in both groups at study exit (placebo: −0.6 kg [−1.3 to +0.3], *p* = 0.03; exenatide‐ER: −0.2 kg [−1.2 to +0.5], *p* = 0.02). Hemoglobin A1c remained unchanged on exenatide‐ER (−0.05% [−6.9 to +2.1]) but increased on placebo (+2.3% [−1.7 to +4.4]; *p* = 0.03).

**Conclusions and Clinical Importance:**

Exenatide‐ER contributed to the maintenance of glycemic control as reflected by hemoglobin A1c but did not affect remission duration. Management might have contributed to the extended remission duration.

**Abbreviations**
ALIVE: Agreeing Language in Veterinary Endocrinology
BG: blood glucose
DM: diabetes mellitus
exenatide‐ER: exenatide extended‐release
GLP‐1: glucagon‐like peptide‐1
GLP‐1RA: GL‐P1 receptor agonist
SC: subcutaneous
sFructosamine: serum fructosamine concentration
SGLT2i: sodium‐glucose cotransporter‐2 inhibitor

## 1 Introduction

Treatment of diabetes mellitus (DM) in cats relies upon the administration of exogenous insulin or a sodium‐glucose cotransporter‐2 inhibitor (SGLT2i), along with dietary modification and mitigation of insulin‐resistant disorders [1]. About 30% of insulin‐treated cats regain sufficient β cell function to sustain euglycemia without the need for exogenous insulin and therefore enter a state of remission [2]. It is assumed that the reversal of glucose toxicity enables pancreatic β cells to resume function. Cats in remission, however, remain metabolically abnormal [3, 4] and the duration of remission after insulin therapy is usually just a few months [2]. This is presumably because of progressive deterioration of insulin secretion capacity or of insulin sensitivity, or both [5]. Whether the same is true for cats receiving SGLT2i's is currently unknown.

Glucagon‐like peptide‐1 (GLP‐1) is a hormone secreted from the small intestine in cats in response to nutrients within the bowel lumen [6]. Its effect in the cat pancreas is glucose‐dependent: GLP‐1 does not stimulate insulin secretion when blood glucose concentrations (BG) are normal, but when BG increases, GLP‐1 potentiates insulin secretion and inhibits the release of glucagon [6, 7, 8]. Therefore, the effect of GLP‐1 on insulin secretion in a euglycemic cat should be minimal, and an overdose of GLP‐1 or its analogs is unlikely to induce hypoglycemia. Studies in people and rodent models show that chronic administration of GLP‐1 receptor agonists (GLP‐1RA) preserves and even increases β cell mass and the capacity to secrete insulin [9, 10].

Exenatide is a GLP‐1RA that is widely used to treat people with non‐insulin‐dependent type 2 DM. Its extended‐release formulation (exenatide‐ER) was the first FDA‐approved once‐weekly injection for the treatment of this disorder. In a long‐term clinical trial in human patients with type 2 DM, a once‐weekly injection of exenatide‐ER was more effective in controlling hyperglycemia than once‐daily insulin glargine, with fewer side effects such as hypoglycemia and weight gain [11]. Importantly, treatment with exenatide‐ER maintained these diabetic people in an insulin‐independent state with near‐euglycemia for years and helped to curb weight gain [11]. GLP‐1RA are also reported to improve the survival and function of transplanted pancreatic islets in type 1 DM [12, 13].

The action of GLP‐1 and GLP‐1RA appears to be similar in cats as in other species [14]. It is not yet known if, independent of BG concentrations, GLP‐1 stimulates β cell proliferation and survival and increases β cell mass in cats as it does in other species [9, 10]. However, it is possible that GLP‐1 might curb appetite and so, help with weight loss in cats [15, 16]. Therefore, we hypothesized that GLP‐1RA might prolong the duration of remission in cats.

The objective of this study was to compare the effect of once‐monthly injection of exenatide‐ER to placebo on the duration of remission (insulin independence) in cats with DM.

## 2 Materials and Methods

### 2.1 Cats

Client‐owned diabetic cats in remission were recruited for this study. Cats were included if they were previously diagnosed with DM and were treated with insulin (any type) for at least 2 weeks. Diagnosis of DM and definition of diabetic remission were consistent with Project ALIVE (Agreeing Language in Veterinary Endocrinology) [17]. Cats were only included if their diagnosis of remission was recent, that is, insulin therapy had been discontinued at least four but no more than 12 weeks before entry into the study, and if there was no evidence of diabetic relapse. Cats were also only included if they were routinely fed a low‐carbohydrate diet (e.g., < 25% DM, < 15% ME, < 5 g/100 kcal). Study candidates were excluded if they had: (1) A recent administration (previous 3 months) of an oral hypoglycemic agent or GLP‐1 analog; (2) A history of glucocorticoid administration in the 3 months before diagnosis of DM; (3) Concurrent disease that might require exogenous steroid administration, reduce life expectancy to < 24 months, or require intensive management, for example, gastrointestinal, pulmonary, cardiac, hepatic, neoplastic, or renal disorders (CKD IRIS Stage 3 or higher); (4) Suboptimal body condition score (BCS ≤ 3/9); (5) Overt evidence of peripheral neuropathy or hypersomatotropism; (6) Untreated hyperthyroidism.

### 2.2 Study Design

**FIGURE 1:**
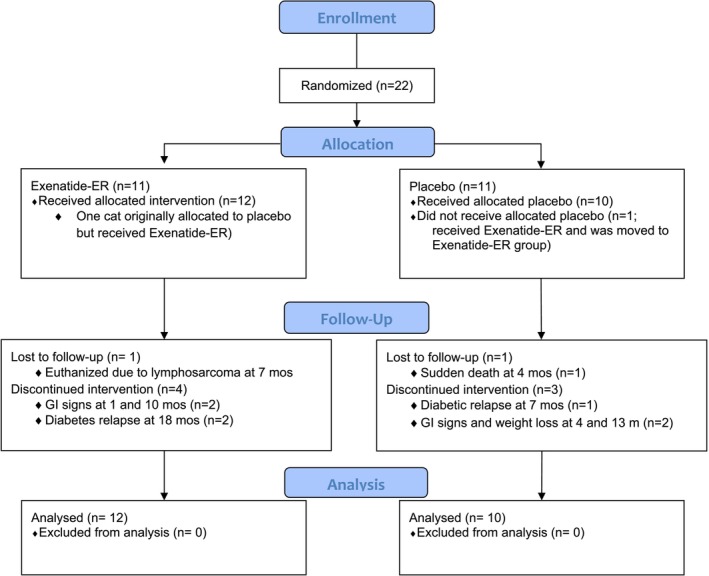
Study timeline.

Study timeline is depicted in Figure 1. For each cat, prescreening to meet inclusion criteria (day 0) included a complete blood count, serum biochemistry panel, total thyroxine (TT4), and verification of remission status [17] (either by serum fructosamine concentration [sFructosamine] or negative urine glucose on more than one occasion on naturally voided urine acquired in a home environment at least 2 days after a stressful event).

This was a single‐blinded, placebo‐controlled study. Random group assignment throughout all centers that participated in this study was facilitated by a single website: Once logged in, the clinician was prompted to enter basic information (patient name, age, sex, body weight, duration of remission). If the cat qualified for enrollment, the clinician received an automatic email assigning the cat to a treatment group. Cats were randomized to receive one of two treatments: Exenatide‐ER (Bydureon, AstraZeneca LTD. Cambridge, UK; exenatide‐ER) at 0.13 mg/kg [7] or 0.9% saline (equal volume to Bydureon), SC every 27–32 days for 2 years (total of 23 injections) or until DM relapse. Diabetes relapse was defined as the recurrence of chronic hyperglycemia associated with clinical signs of DM, confirmed either by increased sFructosamine above the reference interval (of the laboratory in which it was measured), or documentation of glucosuria on more than one occasion on naturally voided urine acquired in a home environment at least 2 days after any stressful event. Throughout the study, depending on location and availability, two different formulations of exenatide‐ER were used: Bydureon (2 mg/0.65 mL injection pen) and Bydureon BCise (2 mg/0.8 mL injection pen). Both injection pens were designed to deliver the full 2 mg dose in a single injection. In order to deliver the study dose of 0.13 mg/kg, for both formulations, the pen was first prepared for injection as recommended by the manufacturer, including thorough mixing until complete resuspension of the drug. Then, the entire content of the pen was injected into a sterile syringe or sterile vial. A 1 mL syringe was then used to draw the drug from the sterile vial to administer it SC.

All injections were administered by attending clinicians at the hospital, without disclosing to the owners which treatment was given. Attending clinicians were not blinded to treatment group designation. At each monthly recheck, a thorough history was obtained and a physical exam performed, including measurement of body weight and BCS. Also, at each recheck, BG concentrations were measured. Diabetes relapse was suspected when BG was > 180 mg/dL (> 10 mmol/L), if clinical signs of DM were noted by the owners (polyuria, polydipsia, polyphagia), or if significant unintentional weight loss was recorded (> 10% of body weight at enrollment). When DM relapse was confirmed (as detailed above, using either sFructosamine or urine glucose measured at home) the cat exited the study. If sFructosamine was within the reference interval, or if urine glucose was negative at home, DM was considered to be still in remission, and the cat continued in the study, regardless of clinical signs or other laboratory tests. When available, glycosylated hemoglobin A1c (A1c) was performed monthly but was not used as a criterion for relapse. Throughout the study, all owners were counseled at every recheck on the need for their cat to achieve and maintain optimal body condition and exclusive consumption of a low carbohydrate diet (as defined above). Canned wet cat food was encouraged but not mandated. Meal feeding or ad lib feeding was both permitted. For cases recruited in the USA and Australia (10 in the Placebo group and 10 in the exenatide‐ER group), Purina DM (canned ± dry) was provided by the study free of charge.

Throughout the study, diagnostic tests and procedures that were not part of the study protocol were permitted if judged to be clinically appropriate by the attending clinician. Cats that developed concurrent diseases were to be treated as needed with standard protocols. Developing concurrent diseases after beginning the study was not a reason for withdrawal unless glucocorticoids were required for treatment. Patients were withdrawn from the study if owners did not comply with the study protocol, if unintentional weight loss occurred (> 2% per week), if BCS was < 3/9, if owners reported persistent hyporexia or gastrointestinal signs, or if any other adverse events that might have been associated with drug administration were noted.

A1c was measured with the dried‐blood‐spot mail‐in A1Care test (Baycom Diagnostics, Tallahassee, FL, USA) that was previously validated for use in cats [18]. A1c was not available to all study sites or at all times and was not used as a criterion for relapse. All other laboratory tests were performed in reference laboratories or in clinical pathology laboratories in the authors' respective institutions. Serum fructosamine was measured by the nitroblue tetrazolium colorimetric assay in 28 samples and by a fructosaminase assay in two samples from a single cat. Blood glucose concentrations were measured by a point‐of‐care glucose meter (AlphaTrak2, Blood Glucose Monitoring System, Zoetis, Parisippany, NJ). Urine glucose was measured by urine dipsticks using the glucose‐oxidase method.

### 2.3 Statistical Analysis

Data on the incidence of relapse of DM after remission were scarce when this study was designed, but what was available suggested that without specific treatment, DM would relapse in most cats within a year [3, 19]. The effect of exenatide‐ER on extending remission in diabetic cats had not been reported previously and, therefore, sample size calculation was inherently unreliable. However, given the similarities between DM in people and cats and given the ability of exenatide‐ER to maintain an insulin‐independent state in people for many years, we expected most of the exenatide‐ER group to remain in remission for the duration of the study (24 months). Assuming that at the end of the study the proportion of cats in remission would be ≤ 30% in the control group and ≥ 90% in the exenatide‐ER group, with an alpha error of 5% and 80% power, 12 cats in each group were required to detect a significant difference (Using the fishers exact sample size estimates per Group for two Sided Test, http://hedwig.mgh.harvard.edu/sample_size/fisher/js/fisher.html). Based on interim analysis and lack of difference between the groups, it was later decided to cap the group size to 10 cats.

Statistical analyses were performed using commercial statistical software packages (GraphPad Prism 10.1.1, San Diego, California). Descriptive statistics were generated to characterize the study population. Continuous variables were presented as median and range (minimum and maximum value). Categorical variables (sex, relapse, adverse effects) were described with frequencies and compared between groups with the Fisher exact test. The Shapiro–Wilk test was used to assess normality. For each cat, study exit was recorded regardless of the cause of exit (No relapse after 24 monthly rechecks, DM relapse, unintentional weight loss, GI signs, or other). Differences between day zero and study exit in each group were compared using a paired student *t*‐test (for body weight) and Wilcoxon test (for BCS). Differences between the exenatide‐ER and placebo groups for numerical variables (e.g., age, sFructosamine, A1c, remission time) were compared using the Mann–Whitney test. Both remission time in the study (from inclusion to study exit) and total remission time (from diagnosis of remission until study exit) were calculated and compared between groups. The level of significance was set at *p* < 0.05.

## 3 Results

Twenty‐two spayed/neutered cats, ranging in age from 5 to 13 years, were recruited. One cat that was originally assigned by the randomization software to the placebo group was accidentally started on exenatide‐ER. This resulted in 10 cats participating in the placebo group and 12 in the exenatide‐ER group. All cats presented for monthly rechecks and treatment (whether placebo or exenatide‐ER) as per study protocol throughout the study except for one cat in the placebo group. This cat completed the first 12 rechecks as planned but then, because of a lapse in funding, was not rechecked for another 11 months. Because the cat was still in remission (including a normal BG and fructosamine) at that last recheck, this recheck date was entered as his study exit date.

At inclusion, the groups were not different except in the duration of remission before inclusion (Table 1). In both groups, median body weight decreased (placebo: −0.6 kg [−1.3 to +0.3], *p* = 0.03; exenatide‐ER: −0.2 kg [−1.2 to +0.5], *p* = 0.02) and BCS decreased (placebo: −1[−2 to 0], *p* = 0.02; exenatide‐ER: −1[−2.5 to +1], *p* = 0.03) comparing day zero to study exit. There was no difference in body weight or BCS between groups at entry or study exit (Tables 1 and 2). In the placebo group, one cat relapsed with DM (day 212) and two cats exited the study prematurely (days 121 and 393), while still in remission, because of gastrointestinal signs and weight loss. A fourth cat exited the study because of sudden unexplained death (day 126) but was still in remission at the previous recheck. In the exenatide‐ER group, two cats relapsed with DM (days 553 and 558) and two cats exited the study prematurely because of gastrointestinal signs (days 28 and 315), while still in remission. A fifth cat in the exenatide‐ER group was euthanized after developing lymphosarcoma 7 months after entering the study.

**TABLE 1:** Group characteristics upon study entry. All values presented as median (range). When data were missing, the number of cats with available data are included).

	Placebo *N* = 10	Exenatide‐ER *N* = 12	*p*
Age (years)	11.5 (4–14)	11.5 (8–13)	0.7
Sex (spayed females; neutered males)	4; 6	5; 7	1.0
Body weight (kg)	6.1 (4.5–7.8)	5.9 (4.4–7.5)	0.6
Body condition score (/9)	6.5 (5–8)	6.0 (4.5–7.5)	0.5
Duration of remission before inclusion (weeks)	9 (5–12)	6 (4–9)	0.01
Serum fructosamine (μmol/L)	252 (227–351) *N* = 7	268 (233–347) *N* = 11	0.3
A1c (%)	3.9 (2.1–4.9) *N* = 5	2.8 (1.7–10.5) *N* = 10	0.7

**TABLE 2:** Group characteristics at study exit. All values presented as median (range). When data were missing, the number of cats with available data are included).

	Placebo	Exenatide‐ER	*p*
Body weight (kg)	5.9 (3.4–6.5)	5.7 (3.8–7.3)	0.5
Body condition score (/9)	6.0 (4–7)	5.0 (4–7)	0.4
Duration of remission in study from inclusion (days)	669 (121–721)	662 (28–735)	0.9
Total duration of remission (days)	743 (161–805)	704 (77–763)	0.5
Serum fructosamine (μmol/L)	266 (181–486) *N* = 7	257 (194–369) *N* = 5	1.0
A1c (%)	3.8 (1.8–8.1) *N* = 8	2.7 (1.9–5.1) *N* = 10	0.2

**FIGURE 2:**
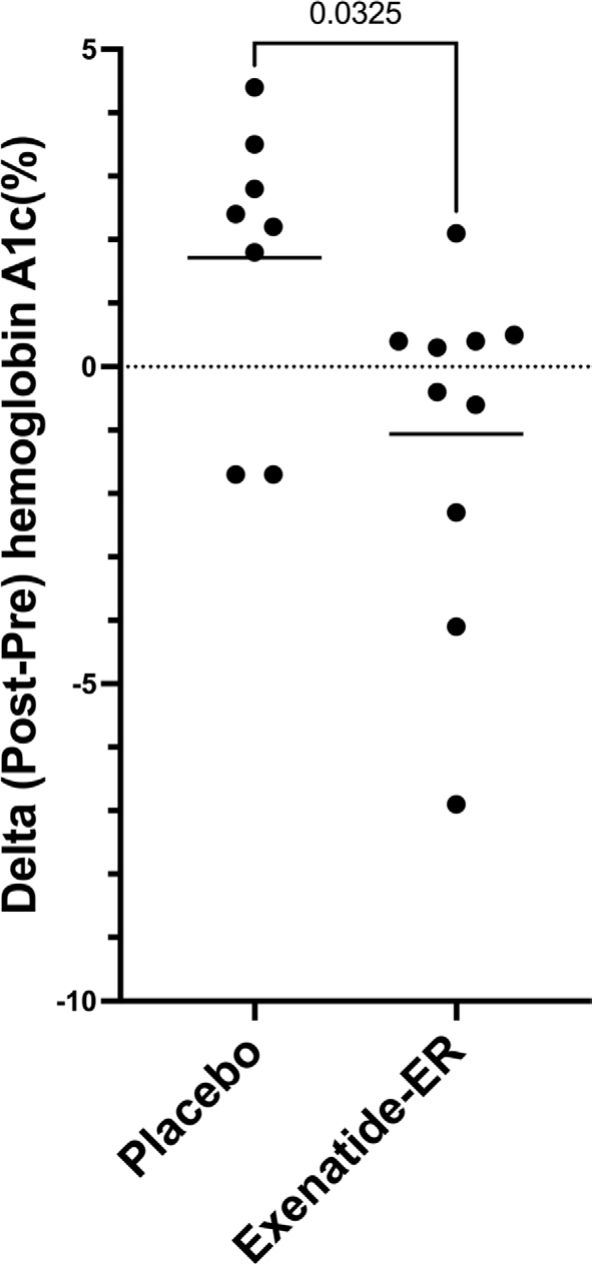
Delta hemoglobin A1c (%) from study entry (pre) to study exit (post) in 22 cats in remission treated with placebo (*n* = 10) or exenatide‐ER (*n* = 12).

For both groups, more than half of the cats (placebo, *n* = 6 and exenatide‐ER, *n* = 7) remained in remission for the full duration of the 2‐year study. When defining the date of exit as the time of relapse, cats in the placebo and exenatide‐ER groups remained in remission for a median (range) of 669 days (121–721) and 662 days (28–735; *p* = 0.9), respectively. After excluding cats that exited the study early for reasons other than relapse of DM, the median (range) of total remission time was 745 days (247–805) and 710 days (595–763) in the placebo versus exenatide‐ER group, respectively (*p* = 0.17). Considering both groups as one, the median total remission time was 710 days (77–805) in all cats and 743 days (247–805) when excluding cats that exited the study early for reasons other than DM relapse. In all three cats that relapsed, at the time of relapse, A1c had increased from study entry (5.1% versus 3.0% and 3.1% versus 2.6% in the exenatide‐ER group and 4.3% versus 2.1% in the placebo group). Considering both the cats that maintained remission and the cats that relapsed, A1c did not differ between groups (*p* = 0.2, Table 2) at study exit. After excluding cats that relapsed, there was still no difference in A1c between groups (*p* = 0.2). At study exit, A1c was above the reference interval in 5 out of 8 cats in the placebo group and 3 out of 10 cats in the exenatide‐ER group (*p* = 0.3). When comparing the absolute change from baseline, the placebo group tended to have an increase in A1c% (+2.3% [−1.7 to +4.4]), but the exenatide‐ER group tended toward no change (−0.05% [−6.9 to +2.1], *p* = 0.03, Figure 2).

## 4 Discussion

Our study demonstrates that with frequent monitoring, regular scheduling of nutritional support, and encouragement to achieve and maintain an ideal BCS, most cats in diabetic remission can maintain insulin independence for at least 2 years. Critically, this was observed in cats without overt and treatable causes of insulin resistance (such as hypersomatotropism or exogenous glucocorticoids) other than excess body condition. In contrast to our hypothesis, the study did not show a direct benefit of exenatide‐ER in extending remission over the study period, although treatment with exenatide‐ER did seem to curtail an increase in A1c.

At the inception of this study, some evidence existed regarding factors that contribute to acheiving remission in diabetic cats, but little was known regarding maintenance of remission [2]. In one study, the median duration of remission was only 151 days (< 5 months) [19]. In another study, 30% of cats in remission relapsed within 9 months, however, about a third of cats in that study were treated with glucocorticoids shortly before the diagnosis of DM (making them less likely to relapse with DM once glucocorticoids were discontinued) [3]. Both of these studies were retrospective observational studies: no specific diet, treatment or monitoring were offered once insulin therapy was discontinued. In our study, relapse rates over 2 years were much lower. Even if we consider a “worst case scenario” in which all early exits were considered “relapse,” still no more than 41% of our study population relapsed in 2 years, with a median total duration of remission for all cats of 710 days. This is in contrast to a recent study in which both the diagnosis of DM and subsequent diabetic remission were more lax than those used in our study, previous treatment with glucocorticoids was permitted, and 20% of the population was Burmese cats. Despite the fact that these factors would be expected to contribute to prolonged remission, 40% of cats relapsed within 1 year [20]. After remission was reached in that study, no specific dietary recommendation was made and information on diet during this time was not reported.

Although our study was not designed specifically to assess non‐pharmacologic methods to support remission, it seems likely that the low rate of relapse of DM here was related to the combination of monthly monitoring, the continuous advocacy for maintaining ideal body condition, and the ongoing provision of a low carbohydrate diet. In other words, the “placebo effect” appears to have achieved a substantial beneficial outcome for maintenance of diabetic remission in cats. Placebo‐controlled clinical trials with blinding of participants are an established method of testing medical therapy. Nonspecific effects of the practitioner‐patient interaction are controlled for, rather than investigated [21]. The placebo effect is defined as beneficial changes in the placebo arm of a study compared with a natural history/no treatment arm, whereas the nocebo effect identifies detrimental changes in the placebo arm compared with natural history/no treatment [22]. The beneficial effects of practitioner‐patient interaction are now recognized to have considerable influence in weight loss and diabetes management in people and have led to the development of strategies such as nudge interventions in these fields [22, 23]. The situation is likely similar for cats and their caretakers. Effective communication between the veterinary team and the cat's owner and regular scheduling of nutritional support and encouragement to achieve and maintain an ideal BCS are recommended for prevention and management of obesity in cats [24]. Ongoing monitoring is also a critical factor in the management of cat DM [1].

Again considering the “worst case scenario” in which all early exits were considered “relapse,” there would still be no difference in remission duration and the frequency of relapse between the placebo and exenatide‐ER groups (40% and 42% respectively). Based on these data, no effect of exenatide‐ER duration of remission of DM in cats was observed. It is of course possible that exenatide‐ER has a smaller effect on remission duration than could be detected with the sample size in this study. A sample size calculation based on a hypothetical 50% versus 30% relapse frequncy reveals a minimum of *N* = 91 per group to achieve 80% and alpha error < 5%. Considering the multi‐institutional nature of our study and the number of years it took to complete, it was clear that our study would not be able to demonstrate this hypothetical small effect and we therefore decided to terminate the study at its current group size. However, considering the importance of optimizing body condition in achieving remission [5], it is possible that exenatide‐ER might have indirectly contributed in a small way to maintanence of remission in our study by contributing to weight management.

The results of our study indicate that consistent, targeted, practitioner‐client interactions have the potential to provide more value for the maintenance of diabetic remission in cats than treatment with exenatide‐ER. Research to specifically investigate this hypothesis is warranted. The American Diabetes Association recommends nutritional, behavioral, and lifestyle interventions alongside pharmacotherapy with drugs such as GLP‐1RA, medical devices, and bariatric surgery for the prevention and treatment of type 2 DM in people [25]. As the subclinical, pathophysiologic state of cats that have achieved diabetic remission is assumed to be similar to that of prediabetes [5], specific practitioner‐client interventions that are demonstrated to extend diabetic remission might subsequently be used prophylactically to minimize progression to clinical diabetes in cats.

Our study did not demonstrate an additional benefit of exenatide‐ER compared to placebo in long‐term weight loss in cats. We also did not find a difference in the frequency of adverse effects. In the past few years, GLP‐1RA have revolutionized the treatment of obesity and metabolic syndrome in people [26]. By increasing satiety, decreasing appetite, and slowing gastric emptying, GLP‐1RA induce weight loss with an effect that rivals bariatric surgery. In people, their superior safety profile led to their approval as monotherapies in nondiabetic individuals with obesity. Additionally, GLP‐1RA also reduce fat content in the liver and are indicated for the treatment of nonalcoholic hepatic lipidosis in people [26]. Gastrointestinal signs are the most common adverse effects of these agents in people and represent a potential barrier for use [27]. There is some evidence in short‐term studies in cats to support the use of GLP‐1RA to facilitate weight loss in cat obesity, specifically for exenatide, although it was not clear if weight loss was due to increased satiety or to drug‐related gastrointestinal signs [15, 16, 28, 29, 30]. Gastrointestinal side effects might be ameliorated in people by gradual dose escalation and are expected to subside once drug concentrations stabilize [27]. This is the main reason for using exenatide‐ER as a once‐weekly drug in people despite its much longer half‐life. In cats, exenatide‐ER peaks at 3–4 weeks post‐injection and the duration of action is greater than 4 weeks [7], hence our choice to use it as a once‐monthly injection. To minimize gastrointestinal side effects in cats, implantable long‐term drug delivery systems might result in more appropriate dose titration and stabilization of exenatide‐ER than intermittent injections [16, 29].

As in people, our study demonstrates the safety of long‐term administration of exenatide‐ER to euglycemic patients. Although one cat in the exenatide‐ER group was withdrawn shortly after enrollment because of vomiting, it is unclear how often exenatide‐ER causes clinically important adverse effects in cats. Both in our current study and in a previous study comparing exenatide to placebo, the overall frequency of gastrointestinal signs was not different between treatment groups [15]. One limitation of our study is that it was single‐blinded (to owners) but clinicians were aware of the treatment group. In the placebo group, cats might have been withdrawn from the study more readily because of gastrointestinal signs, as those signs likely prompted the attending clinician to assume the cat was suffering from concurrent disease that required diagnosis and treatment. In contrast, clinicians might have been more tolerant of gastrointestinal signs and weight loss in the exenatide‐ER group, potentially attributing these to an adverse reaction to exenatide‐ER.

Our study also had other limitations. It is possible that the lack of differences in desired effect as well as in adverse effects between treatment groups is related to the small sample size in this study. In addition, while cats were randomized into treatment groups, there was a small but significant difference in the duration of remission prior to study entry. To account for that, we analyzed the data both for total remission duration as well as remission duration after study entry and found no difference between groups either way. It is possible that the longer duration of remission before entry in the placebo group might have contributed to the numerically longer total duration of remission in this group compared to the exenatide‐ER group. However, the lack of a statistical difference, together with the fact that the absolute difference is small in comparison to the duration of remission in the study, makes this difference between the groups clinically unimportant in our opinion. Finally, the dose and injection interval chosen for this study were based on a single pharmacology study in healthy cats that showed a sustained insulinotropic effect for a few weeks at a dose of 0.13 mg/kg [7]. It is possible that a higher dose or a shorter injection interval of exenatide‐ER might yield a more obvious effect; however, considering the duration of remission in our control group, that effect would still be likely unobservable over a 2‐year study.

Achieving and maintaining diabetic remission is an important goal for all diabetic cats because it can greatly reduce the treatment burden and improve the quality of life for their owners. What role exenatide‐ER might play in achieving that goal is still unclear. There is evidence that this agent has the potential to provide the same benefits for cats as it does in overweight and diabetic people [7, 15, 16, 29, 30, 31, 32, 33]. It appears that our inability to detect an effect of exenatide‐ER on the duration of remission is the result of a smaller than expected drug effect and/or a greater than expected “placebo effect.” Typically, cats in diabetic remission do not receive any specific medical care and are not routinely monitored. Here, cats in both the placebo and treatment groups were evaluated monthly for 2 years, and their caregivers were consistently reminded of the importance of feeding a low carbohydrate diet and achieving and maintaining ideal BCS. Most cats in this study were also provided a low carbohydrate diet throughout the study. Future studies should examine the effect of practitioner‐client interventions on the duration of remission compared with no intervention at all.

## Disclosure

Authors declare no off‐label use of antimicrobials.

## Ethics Statement

Study design and protocol was approved in each participating institution, The Ohio State University #2015A00000063, University of California, Davis #19584, University of Florida #201910850; 202200000436, University of Copenhagen #2020‐11. Authors declare human ethics approval was not needed.

## Conflicts of Interest

The authors declare no conflicts of interest.
